# Supplementary material for: Analytical Methods for the Characterization of Vegetable Oils
Source: Molecules. 2022 Dec 24;28(1):153. doi: 10.3390/molecules28010153 (PMC9822416; doi:10.3390/molecules28010153)
Supplement: Supplementary file 1 [file molecules-28-00153-s001.zip › molecules-2110699-supplementary.pdf]

**Supplementary materials**

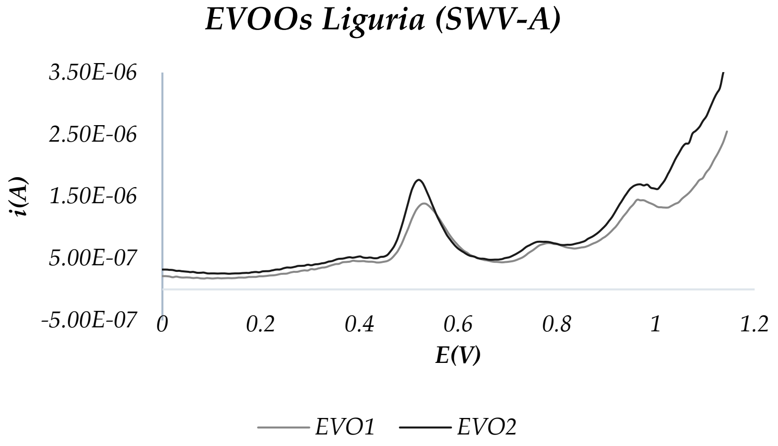


**Figure S01 –** SWV-A of Liguria oils

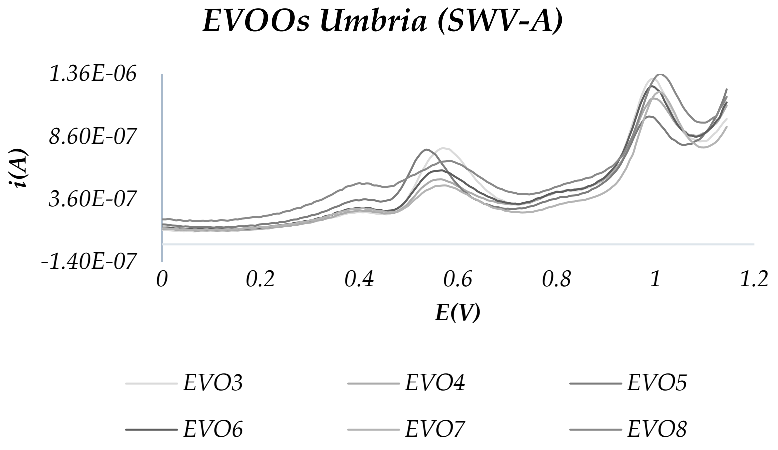


**Figure S02 –** SWV-A of Umbria oils

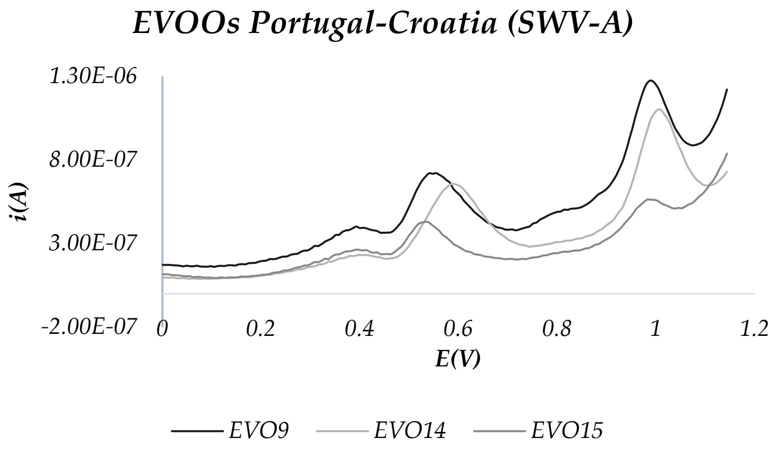


**Figure S03 –** SWV-A of EVOOs from Portugal and Croatia

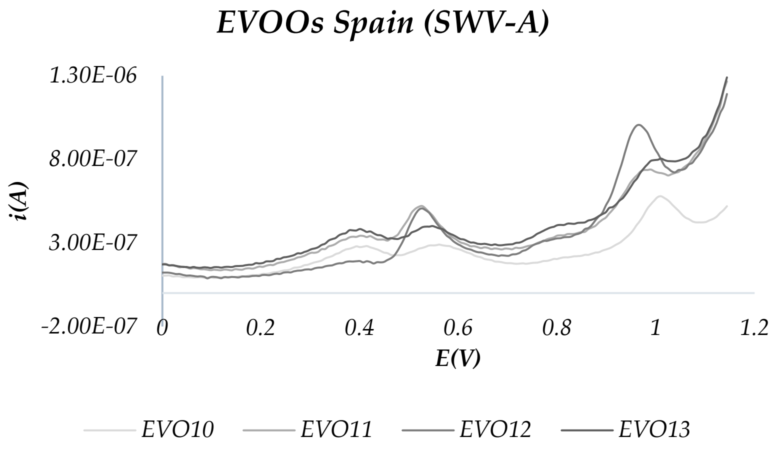


**Figure S04 –** SWV-A of Spain oils

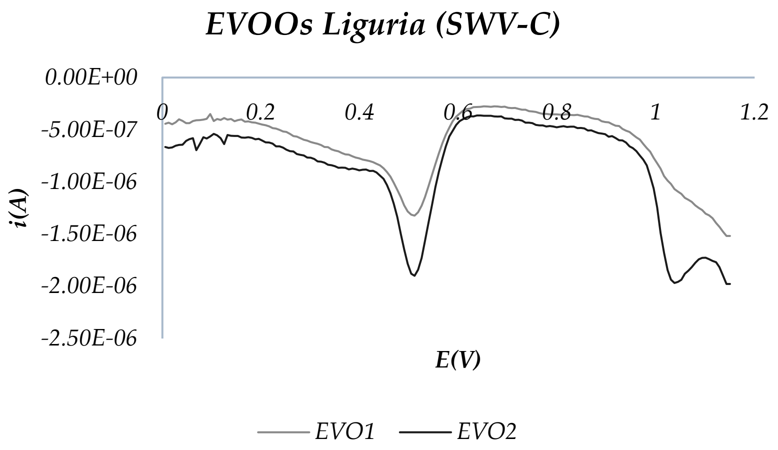


**Figure S05 –** SWV-C of Liguria oils

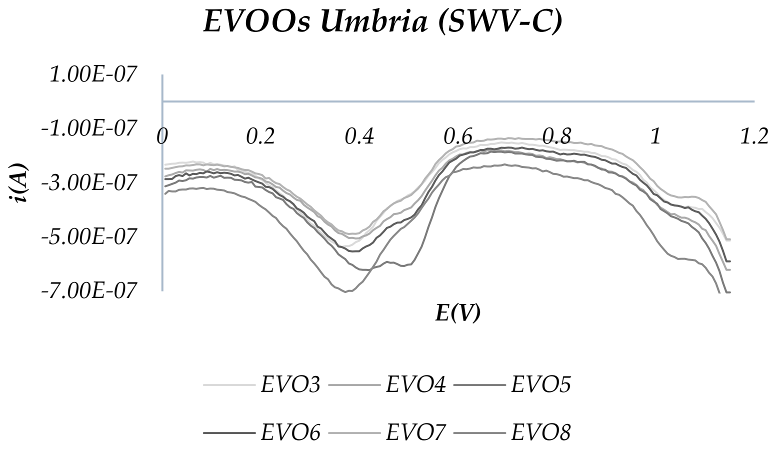


**Figure S06 –** SWV-C of Umbria oils

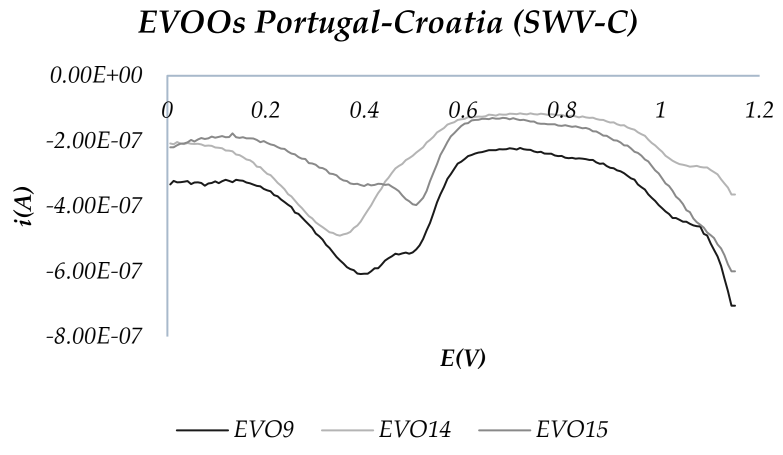


**Figure S07 –** SWV-C of Portugal and Croatia oils

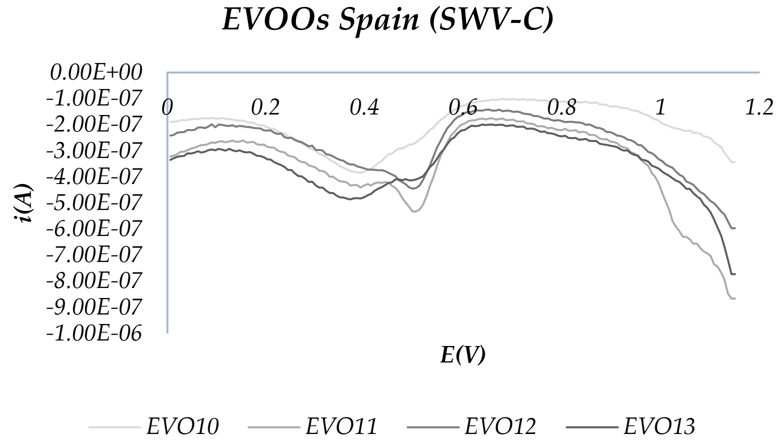


**Figure S08 –** SWV-C of Spain oils

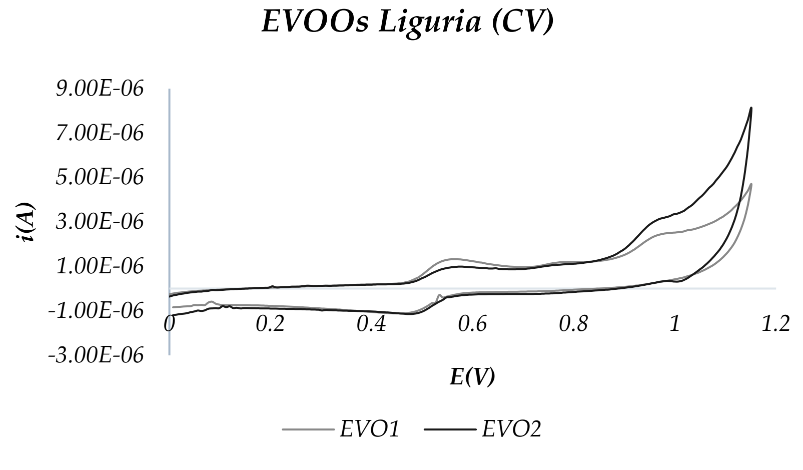


**Figure S09 –** CV of Liguria oils

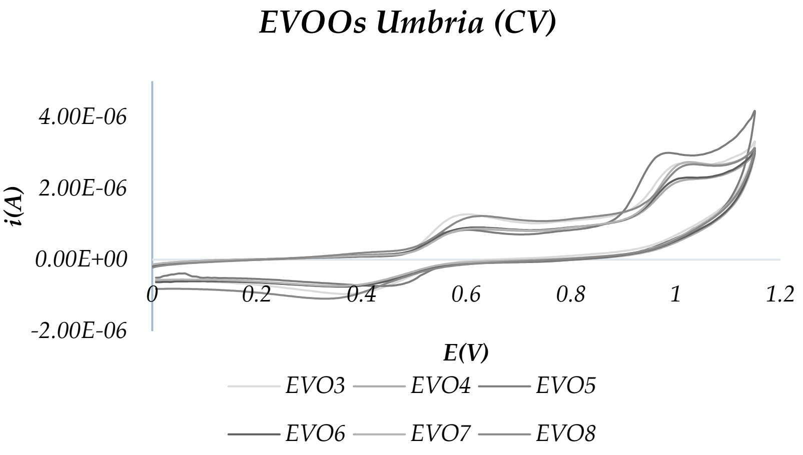


**Figure S10 –** CV of Umbria oils

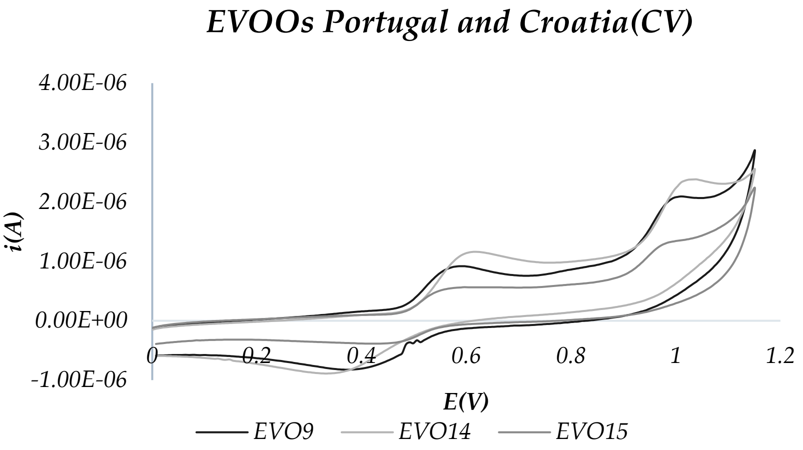


**Figure S11 –** CV of Portugal and Croatia oils

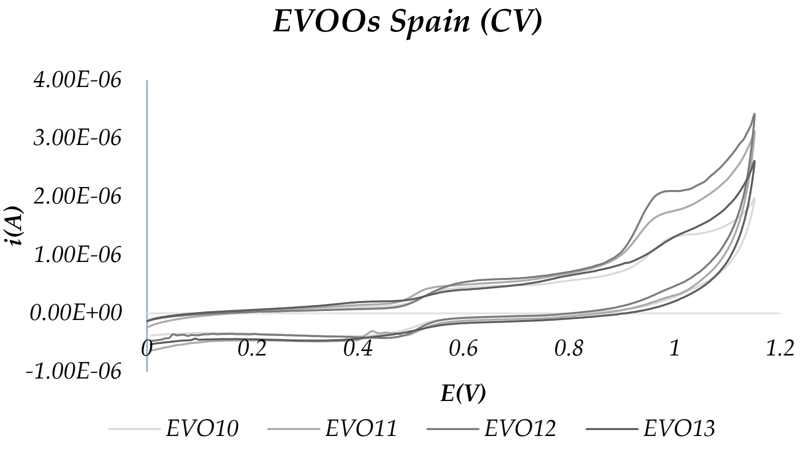


**Figure S12 –** CV of Spain oils

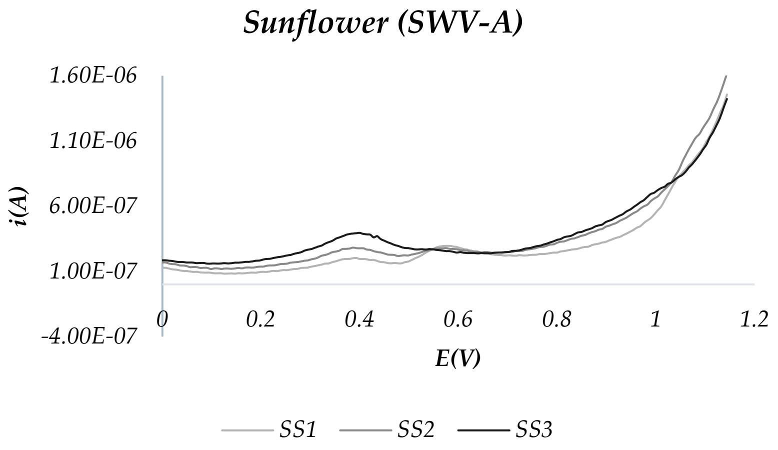


**Figure S13**- SWV-A of sunflower oils

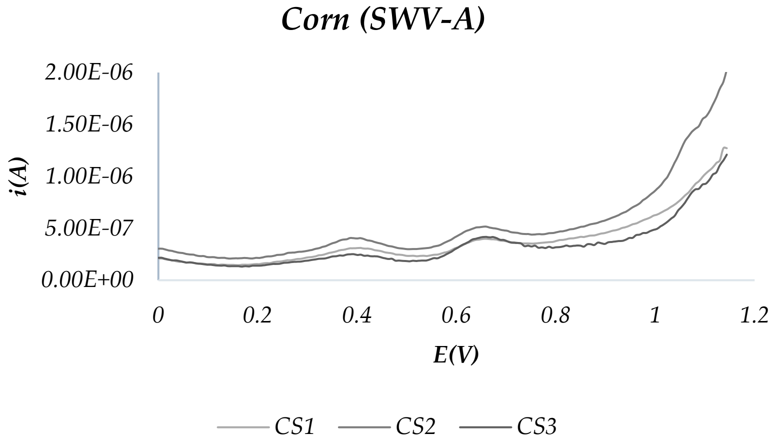


**Figure S14** - SWV-A of corn seed oils

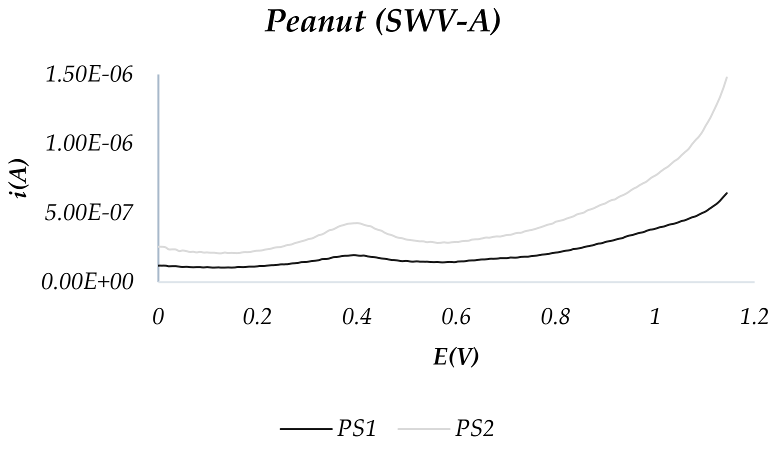


**Figure S15 –** SWV-A of peanut seed oils

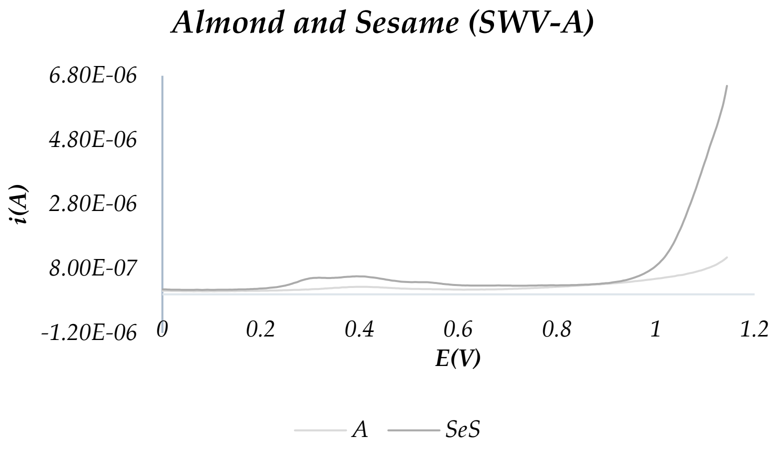


**Figure S16 –** SWV-A of almond and sesame seed oils

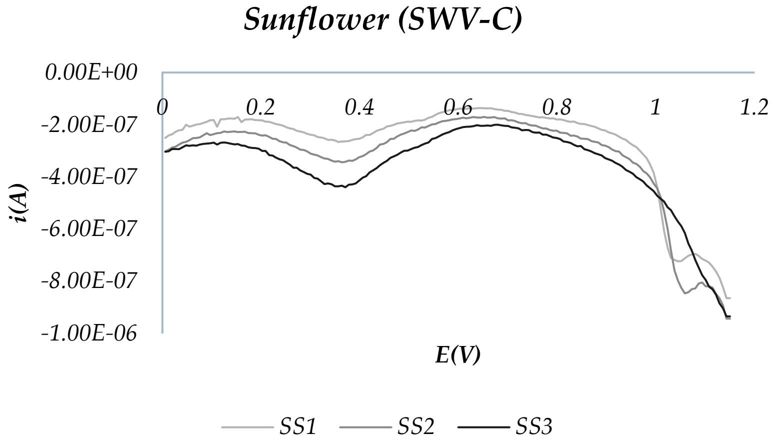


**Figure S17 –** SWV-C of sunflower oils

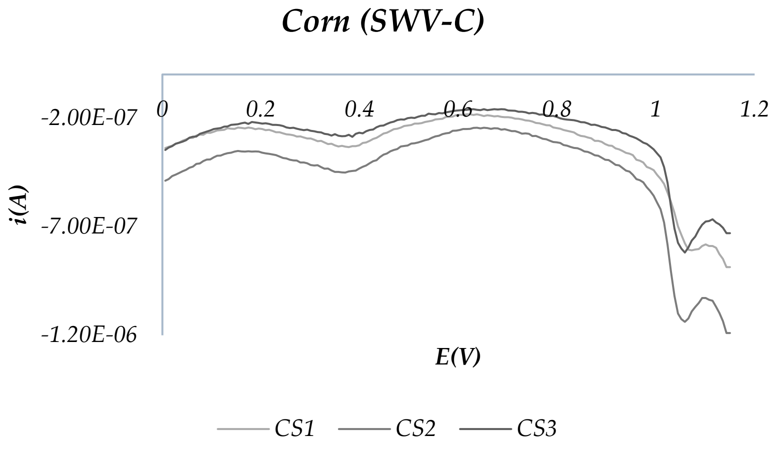


**Figure S18 –** SWV-C of corn seed oils

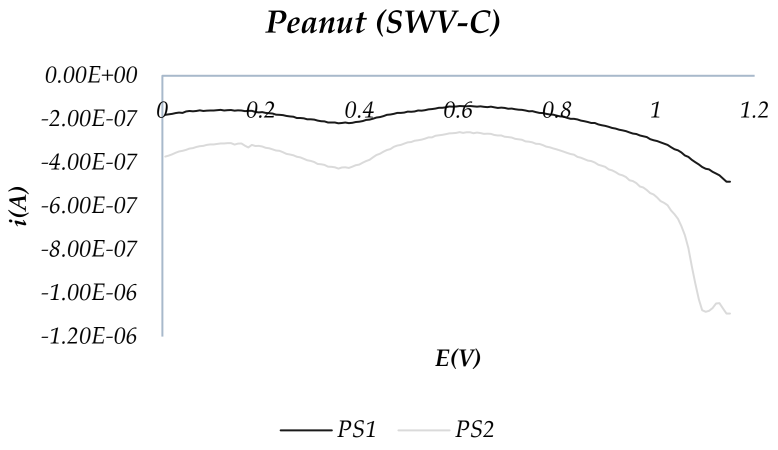


**Figure S19 –** SWV-C of peanut oils

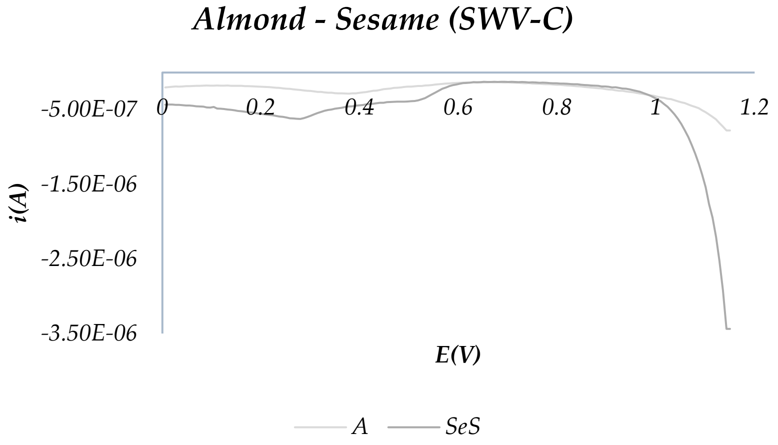


**Figure S20 –** SWV-C of almond and sesame seed oils

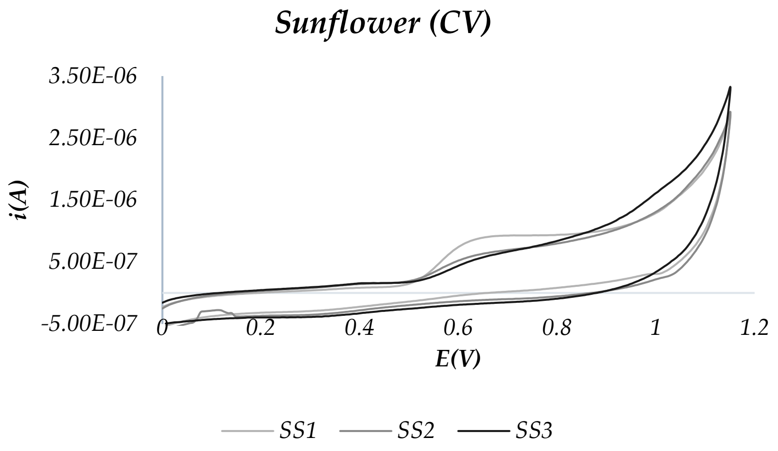


**Figure S21** - CV of sunflower seed oil

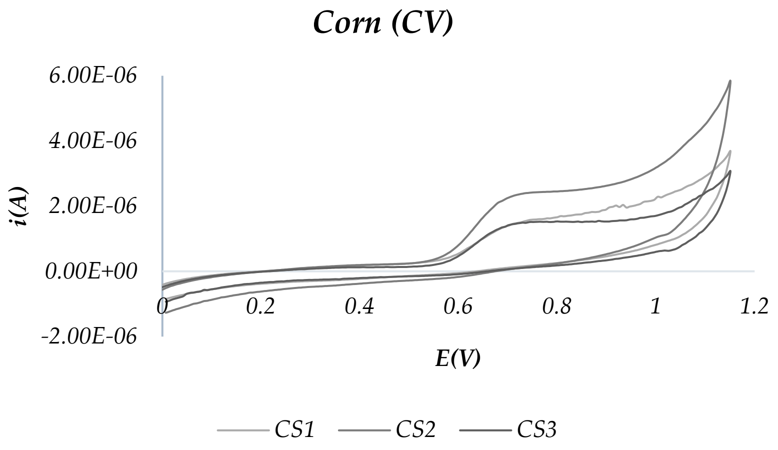


**Figure S22 –** CV of corn seed oils

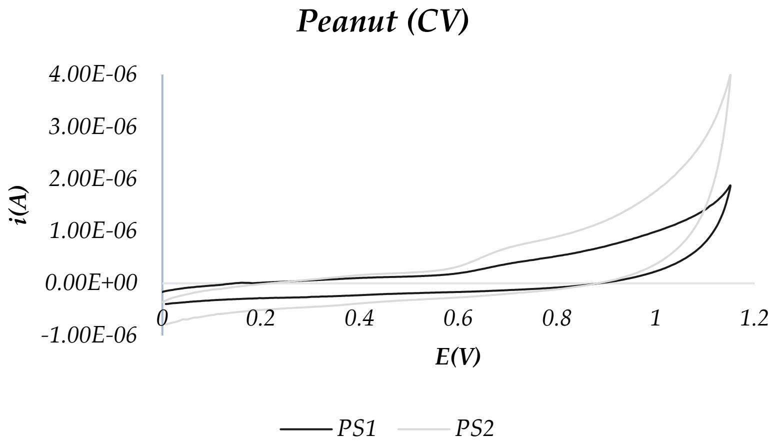


**Figure S23 –** CV of peanut seed oils

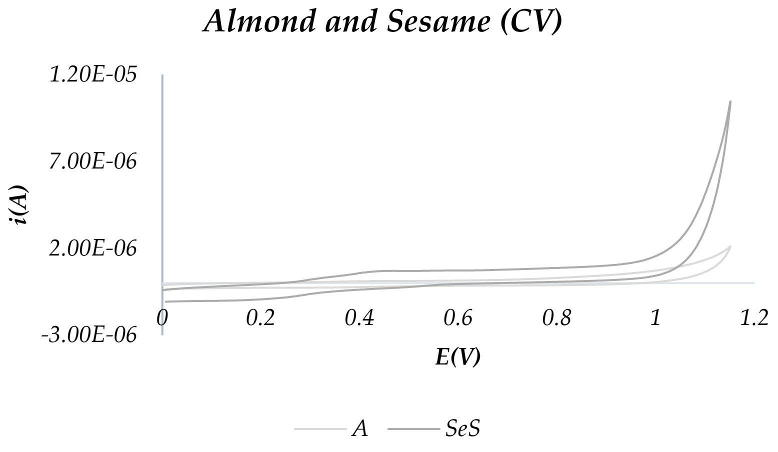


**Figure S24 –** CV of almond and sesame seed oils
